# Prognostic DNA mutation and mRNA expression analysis of perineural invasion in oral squamous cell carcinoma

**DOI:** 10.1038/s41598-024-52745-6

**Published:** 2024-01-29

**Authors:** Su Kyung Kuk, Kitae Kim, Jae Il Lee, KangMi Pang

**Affiliations:** 1 Division of Biomedical Informatics, College of Medicine, Seoul National University, Seoul, Republic of Korea; 2 Department of Molecular Genetics, School of Dentistry and Dental Research Institute, Seoul National University, Seoul, Republic of Korea; 3 Department of Oral Pathology, School of Dentistry and Dental Research, Seoul National University, Seoul, Republic of Korea; 4 Department of Oral and Maxillofacial Surgery, School of Dentistry, Seoul National University, 101, Daehak-ro, Jongno-gu, Seoul, 03080 Republic of Korea

**Keywords:** Oral cancer, Biomarkers

## Abstract

This study analyzed oral squamous cell carcinoma (OSCC) genomes and transcriptomes in relation to perineural invasion (PNI) and prognosis using Cancer Genome Atlas data and validated these results with GSE41613 data. Gene set enrichment analysis (GSEA), gene ontology (GO) and Kyoto Encyclopedia of Genes and Genomes were conducted. We identified 22 DNA mutations associated with both overall survival (OS) and PNI. Among them, *TGFBR1* and *RPS6KA4* mRNAs were overexpressed, while *TYRO3* and *GPR137* mRNAs were underexpressed in PNI patients. Among the 141 mRNA genes associated with both OS and PNI, we found overlap with PNI-related DNA mutations, including *ZNF43, TEX10, TPSD1,* and *PSD3*. In GSE41613 data, *TGFBR1*, *RPS6KA4, TYRO3*, *GPR137, TEX10* and *TPSD1* mRNAs were expressed differently according to the prognosis. The 22 DNA-mutated genes clustered into nervous system development, regulation of DNA-templated transcription, and transforming growth factor beta binding. GSEA analysis of mRNAs revealed upregulation of hallmarks epithelial mesenchymal transition (EMT), TNFα signaling via NF-κB, and IL2 STAT5 signaling. EMT upregulation aligned with the *TGFBR1* DNA mutation, supporting its significance in PNI. These findings suggest a potential role of PNI genes in the prognosis of OSCC, providing insights for diagnosis and treatment of OSCC.

## Introduction

Oral squamous cell carcinoma (OSCC), one of the ten most common cancers worldwide, is regarded as the main cause of death from oral diseases. Treatment of OSCC is mainly based on surgical resection with or without adjuvant treatment. The indication for adjuvant treatment is determined by histopathological data of resected tissue, including status of margins, depth of invasion, vascular/neural invasion, bone invasion, nodal status, and pTNM staging^1^. Among them, perineural invasion (PNI) is defined as the presence of tumor cells around more than 33% of the nerve circumference of a nerve exceeding 1 mm in diameter^2^. PNI has been a reported independent survival factor, in addition to AJCC-TNM tumor staging. Patients with PNI have a high recurrence rate and poor prognosis for several tumors, such as pancreatic, gastric, biliary tract, prostate, head and neck, colorectal, and cervical cancer^3^. The presence of PNI in OSCC of the tongue predicts worse disease-specific survival, with distant recurrence as the most common pattern of failure, while not predictive of local and regional recurrence^4^.

Numerous studies have been conducted to elucidate the key factors related to PNI. An immunohistochemical analysis has demonstrated that within OSCC, cases exhibiting PNI manifest a higher frequency of expression of nerve growth factor and tyrosine kinase A when compared to OSCC cases devoid of PNI^5^. Transcriptomic studies in HNSCC indicated that genes associated with PNI were related to muscle differentiation/function, with Ark/Protein kinase B and mammalian target of rapamycin (mTOR) kinases being increasingly activated^6^. Fibroblasts were also reported to play an important role in PNI through weighted gene co-expression network analysis^7^. However, there were few studies evaluating both DNA mutations and mRNA expression in HNSCC. Considering both FAT1 mutation and mRNA expression together, Kim et al. found that the changes in FAT1-related genes were significantly related to patient prognosis and response of radiation therapy in HNSCC patients^8^. Transcript alterations often stem from somatic changes within cancer genomes and exploring the RNA alterations within the genomic framework provides a valuable resource for uncovering genes and mechanisms functionally associated with cancer^9^. Whole genome, transcriptome and methylome profiling has been reported to improve the discovery of actionable target in high-risk pediatric cancer^10^. In clinical practice it is crucial to identify patients at a higher risk of developing progressive disease for the timely administration of appropriate treatments to reduce the morbidity associated with disease progression. Simultaneously, it is essential to avoid over treatment^11^. Therefore, this study evaluated the prognostically important genes and mechanisms involved in the PNI of OSCC using whole exome somatic mutation and mRNA sequencing data.

## Patients and methods

### Patient population

Data of patients with OSCC who had undergone surgical resection were obtained from the Cancer Genome Atlas (TCGA) Portal. Patients with tumors located in the pharynx, hypopharynx, larynx, and oropharynx were excluded from the study. Among the initial 320 OSCC patients, 15 patients with a prior malignancy, 21 patients with an unknown stage, and 56 patients with unknown perineural invasion status were excluded. This left a total of 228 patients for analysis. Whole exome somatic mutation data from 224 patients and mRNA sequencing data from 218 patients were used in the study. The Genomic Data Commons' (GDC) recommended clinical elements and survival outcome data was utilized^12^. Additionally, GSE41613 (https://www.ncbi.nlm.nih.gov/geo/query/acc.cgi?acc=GSE41613) served as a validation set in the gene expression omnibus (GEO) datasets. Out of the 97 HPV-negative OSCC patients, 96 were analyzed and verified, excluding 1 patient with an unknown treatment status. This study adhered to the GDC Data Use Agreement and the study-specific Data Use Certification Agreement available in the database of Genotypes and Phenotypes (dbGaP).

### Somatic mutation and mRNA sequencing data

Whole exome somatic mutation and mRNA sequencing (RNA-Seq) data, released on March 29, 2022, were downloaded from the TCGA data portal. Somatic variants were obtained in the mutation annotation format (MAF). Whole exome sequencing was performed using the Illumina Genome Analyzer IIX platform. Loss-of-function mutations were defined as nonsense mutations, frameshift indels, and splice-site mutations. RNA-Seq by Expectation Maximization (RSEM) data were generated using the Illumina HiSeq 2000 RNA-Seq platform (version 2), and normalized RSEM data were used for estimating mRNA expression.

### Statistical analysis

Univariate and multivariate Cox regression analyses were conducted to estimate Hazard Ratios (HRs) and their corresponding 95% confidence intervals (95% CIs) for overall survival after adjusting for all major clinical variables. The underlying assumptions of the Cox models were verified through regression diagnostics using Schoenfeld and df beta residuals. Survival analysis was performed using the Kaplan–Meier method, and overall survival was compared between two groups using the log-rank test. Fisher's exact tests were employed to investigate differentially mutated genes. For each gene, a 2 × 2 contingency table of mutation frequencies was constructed for different stage groups, and Fisher's exact test was used to identify genes with significant differences in mutation frequency. Furthermore, differential mRNA expression was assessed through DESeq2 analyses of differentially expressed genes (DEGs) using transcripts per million mapped reads (TPM) values. DESeq2 analyzes differential expression based on a model using the negative binomial distribution. All statistical analyses were performed using R software (version 4.2.2).

### Gene set enrichment and over-representation analysis

Gene Set Enrichment Analysis (GSEA) was conducted based on the presence or absence of PNI in OSCC patients. GSEA calculates the enrichment score (ES) by analyzing the ranked list of genes, incrementing a running-sum statistic when a gene is in the gene set and decrementing it when it is not. The ES reflects the degree to which a gene set is overrepresented at the top or bottom of the ranked list of genes.

The Kyoto Encyclopedia of Genes and Genomes (KEGG) is a database resource used to understand high-level functions and utilities of biological systems. The KEGG mapper online tool was employed to perform KEGG analysis. Additionally, the Database for Annotation, Visualization, and Integrated Discovery (DAVID) from the National Institutes of Health in Bethesda, MA, was used for Gene Ontology (GO) enrichment analysis.

## Results

### Patients

In this study, we analyzed 224 patients from TCGA dataset and 96 patients from GEO dataset with primary OSCC were analyzed (Table S1 and S2). The TCGA patient cohort had an average age of 61.7 years (range, 19–90 years), consisting of 153 males and 71 females. Among the 224 TCGA patients, 103 had passed away, and the median survival time was 36.4 months. The mean follow-up period for the surviving patients was 36 months. Disease-specific and overall survival analyses were performed using the GEO dataset as the validation set, revealing that 29 and 50 out of the 96 patients died, respectively. Multivariate Cox proportional hazard regression analysis was conducted on all significant clinical variables. Stage, perineural Invasion (PNI), lymphovascular invasion, and treatment modality all yielded statistically significant results (Table S3).

### Prognostic DNA mutations in perineural invasion

The DNA mutation status of two groups, categorized by the presence and absence of PNI, was analyzed using Fisher's exact test. The results revealed that 59 genes exhibited differential mutations (*p* < 0.01). Among them, 41 genes (*TP53, CDC20, IGKV1-16, TSPOAP1, SEC16A, PLCB1, MCM3AP, NUP210L, VCAN, TOP3A, PTPN20, PGM3, PPP2R3A, CAMSAP2, IRS4, BAIAP2L1, TRIM42, OXA1L, ALKBH1, FRMPD1, MARF1, FLI1, GPR137, PCDHGC4, SMAD9, EML3, KSR1, PLEKHG1, TRIP12, CDHR3, NVL, PLOD3, DCST2, EHHADH, PLCG1, TCF4, ESYT1, GPRC6A, PKP2, PRAMEF6, TRAF2*) were significantly enriched in the group with PNI. The other 18 genes (*TRBV7-6, KRT80, LIN28A, MROH6, XRCC4, UBTFL1, CC2D2A, LMOD2, MLLT1, MAB21L3, NAA16, FIP1L1, YTHDC1, ADAMTSL4, ZNF474, CNPPD1, TPSD1, LSR*) were significantly enriched in the group without PNI.

**Figure 1:**
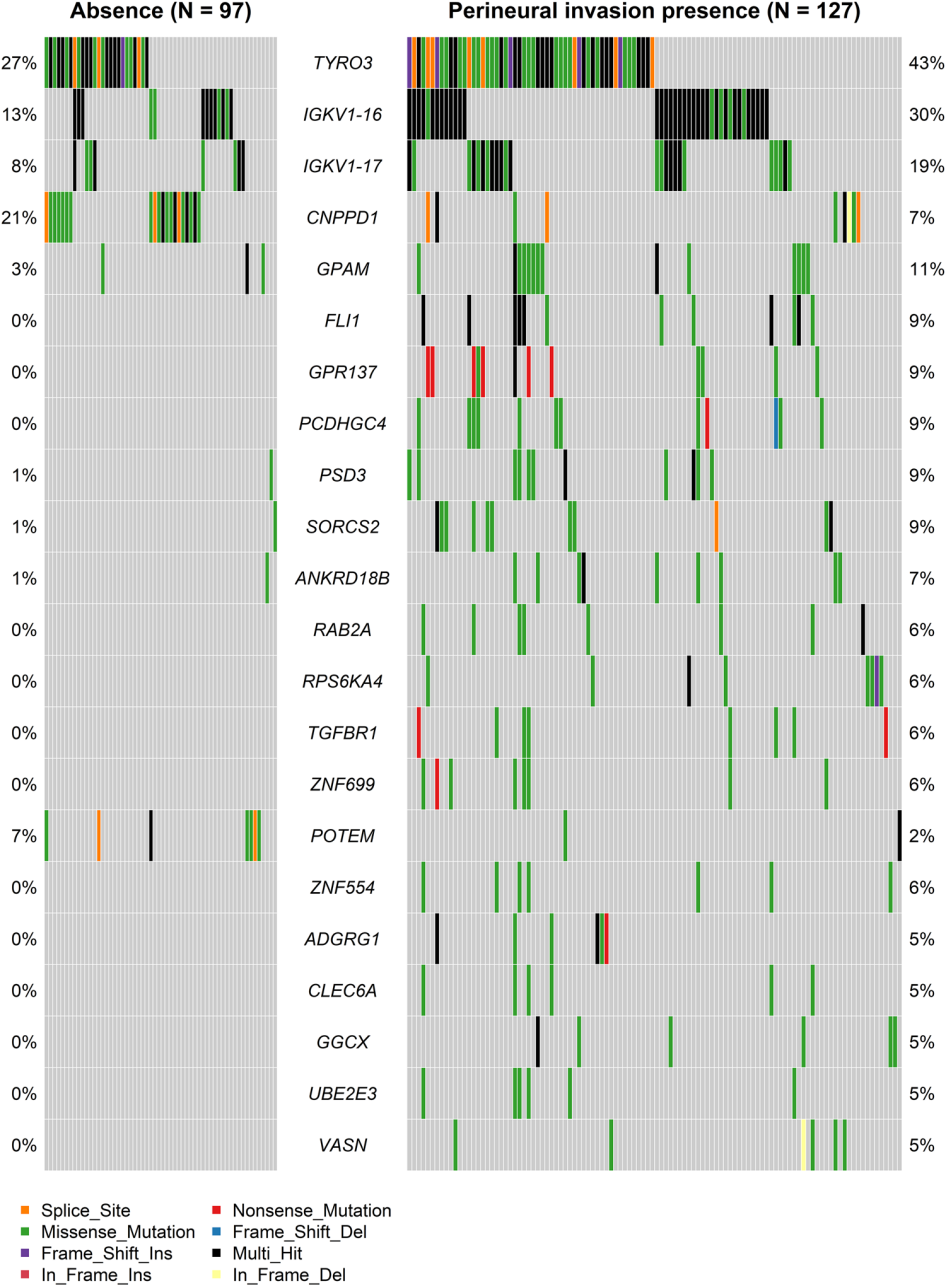
Oncoplots displaying the profiles of 22 prognostically significant genes in oral squamous cell carcinoma patients with perineural invasion (N = 224). These genes demonstrated significant DNA mutations in cases with perineural invasion, as determined by Fisher's exact test, and exhibited prognostic significance in univariate Cox analyses. Furthermore, *ANKRD18B, ZNF699, UBE2E3, ZNF554, VASN, IGKV1-17, PCDHGC4, GPAM, SORCS2, FLI1, RAB2A,* and *CNPPD1* were identified as prognostically significant in multivariate Cox analysis (Table 1).

A total of 388 differentially DNA mutated genes (*p* < 0.05) were identified in the previous comparative analysis. Among them, 22 genes were found to be significantly relevant (p < 0.05; Table S3) for OSCC prognosis. A total of 19 genes (*ANKRD18B, ZNF699, UBE2E3, ZNF554, VASN, IGKV1-17, CLEC6A, PCDHGC4, GPAM, SORCS2, FLI1, ADGRG1, PSD3, TGFBR1, GGCX, IGKV1-16, RAB2A, TYRO3, GPR137*) were enriched in the group with PNI, and their Hazard Ratios (HRs) were higher than 1.0. However, among the PNI-enriched group, *RPS6KA4* was the only gene with HRs below 1.0. Conversely, 2 genes (*CNPPD1, POTEM*) were enriched in the group without PNI and had HRs below 1.0. Oncoplots of the 22 genes were generated for all 224 OSCC patients, as shown in Fig. 1.

These 22 genes were adjusted for all major clinical variables through multivariate Cox regression. A total of 11 genes enriched in the group with PNI (*ANKRD18B, ZNF699, UBE2E3, ZNF554, VASN, IGKV1-17, PCDHGC4, GPAM, SORCS2, FLI1, RAB2A*; Table 1) exhibited significant adjustments for age, stage, lymphovascular invasion, and treatment modality in multivariate Cox analyses. However, *CNPPD1*, enriched in the group without PNI, showed significant results in multivariate Cox analyses (Table 1). *RPS6KA4*, *POTEM*, and *TYRO3* showed a marginal trend toward significance in multivariate Cox analyses (*p* = 0.06, 0.06, and 0.09; HR = 0.15, 0.16, and 1.43, respectively).

**Table 1:** Multivariate Cox regression models of prognostically significant genes in perineural invasion of oral squamous cell carcinoma (The Cancer Genome Atlas patients, N = 224).

Variable	ANKRD18B^a^	ZNF699^a^	UBE2E3^a^	ZNF554^a^	VASN^a^	IGKV1-17^a^
	HR (*P*)	HR (*P*)	HR (*P*)	HR (*P*)	HR (*P*)	HR (*P*)
Gene						
Wild type vs mutant type	2.62 (0.01^c^)	3.07 (0.01^c^)	3.03 (0.02^c^)	2.81 (0.02^c^)	2.73 (0.03^c^)	2.43 (0.0009^c^)
Age	1.02 (0.06)	1.02 (0.03^c^)	1.02 (0.03^c^)	1.02 (0.03^c^)	1.02 (0.03^c^)	1.02 (0.04^c^)
Sex	0.92 (0.71)	0.95 (0.83)	0.97 (0.91)	0.96 (0.84)	0.88 (0.56)	0.87 (0.53)
Race						
White vs nonwhite	1.69 (0.11)	1.71 (0.1)	1.66 (0.12)	1.71 (0.1)	1.72 (0.1)	1.83 (0.06)
white vs unknown	1.06 (0.91)	0.95 (0.93)	0.96 (0.94)	1.29 (0.63)	1.29 (0.63)	1.55 (0.41)
Tumor site						
Tongue vs other sites^b^	1.11 (0.69)	1.03 (0.89)	1.06 (0.81)	1.07 (0.79)	1.16 (0.57)	1.09 (0.73)
Tongue vs unknown	1.16 (0.58)	1.14 (0.62)	1.19 (0.52)	1.21 (0.48)	1.18 (0.55)	1.07 (0.81)
AJCC stage						
Stage 1, 2 vs 3, 4	2.34 (0.01^c^)	2.5 (0.002^c^)	2.49 (0.003^c^)	2.59 (0.002^c^)	2.47 (0.004^c^)	2.68 (0.001^c^)
Lymphovascular invasion						
Absence vs presence	1.94 (0.004^c^)	1.64 (0.03^c^)	1.66 (0.03^c^)	1.72 (0.02^c^)	1.83 (0.01^c^)	1.78 (0.01^c^)
Absence vs unknown	1.06 (0.88)	0.93 (0.84)	0.93 (0.84)	0.97 (0.93)	1.01 (0.97)	0.92 (0.81)
Treatment						
Surgery vs surgery + RT	0.54 (0.03^c^)	0.54 (0.03^c^)	0.55 (0.04^c^)	0.52 (0.02^c^)	0.53 (0.03^c^)	0.54 (0.03^c^)
Surgery vs surgery + CCRT	0.74 (0.3)	0.67 (0.18)	0.71 (0.24)	0.68 (0.19)	0.73 (0.27)	0.8 (0.43)
Surgery vs unknown	1.77 (0.11)	2.08 (0.04)	2.1 (0.03)	1.89 (0.07)	1.89 (0.07)	2.45 (0.01)

Variable	PCDHGC4^a^	GPAM^a^	SORCS2^a^	FLI1^a^	RAB2A^a^	CNPPD1^a^
	HR (*P*)	HR (*P*)	HR (*P*)	HR (*P*)	HR (*P*)	HR (*P*)
Gene						
Wild type vs mutant type	2.21 (0.04^c^)	2.36 (0.009^c^)	3.11 (0.004^c^)	2.96 (0.004^c^)	4.1 (0.001^c^)	0.35 (0.02^c^)
Age	1.02 (0.01^c^)	1.03 (0.01^c^)	1.02 (0.03^c^)	1.02 (0.04^c^)	1.02 (0.02^c^)	1.02 (0.01^c^)
Sex	0.96 (0.84)	0.99 (0.97)	0.88 (0.58)	0.9 (0.64)	0.92 (0.7)	0.96 (0.85)
Race						
White vs nonwhite	1.68 (0.11)	1.7 (0.1)	1.75 (0.09)	1.72 (0.09)	1.65 (0.12)	1.44 (0.26)
White vs unknown	1.34 (0.58)	1.07 (0.91)	1.31 (0.61)	1.03 (0.95)	1.29 (0.63)	1.42 (0.51)
Tumor site						
Tongue vs other sites^b^	1.02 (0.93)	1.0 (0.99)	1.12 (0.65)	1.07 (0.78)	1.15 (0.58)	0.95 (0.84)
Tongue vs unknown	1.1 (0.73)	1.06 (0.83)	1.06 (0.83)	1.18 (0.55)	1.24 (0.44)	1.15 (0.61)
AJCC stage						
Stage 1, 2 vs 3, 4	2.63 (0.002^c^)	2.6 (0.002^c^)	2.68 (0.001^c^)	2.87 (0.001^c^)	2.65 (0.002^c^)	2.5 (0.003^c^)
Lymphovascular invasion						
Absence vs presence	1.76 (0.01^c^)	1.63 (0.04^c^)	1.85 (0.01^c^)	1.75 (0.01^c^)	1.73 (0.02^c^)	1.76 (0.01^c^)
Absence vs unknown	0.95 (0.89)	0.95 (0.88)	0.98 (0.96)	0.92 (0.81)	0.83 (0.61)	0.9 (0.77)
Treatment						
Surgery vs surgery + RT	0.54 (0.04^c^)	0.53 (0.03^c^)	0.47 (0.01^c^)	0.51 (0.02^c^)	0.47 (0.01^c^)	0.5 (0.02^c^)
Surgery vs surgery + CCRT	0.77 (0.35)	0.78 (0.38)	0.69 (0.19)	0.7 (0.22)	0.68 (0.19)	0.75 (0.32)
Surgery vs unknown	1.71 (0.14)	1.91 (0.07)	1.87 (0.08)	2.13 (0.03)	2.16 (0.03)	1.95 (0.05)

*AJCC* American Joint Committee on Cancer, *RT* radiotherapy, *CCRT* concurrent chemotherapy and radiotherapy, *HR* hazard ratio.

^a^The statistical significance of fitted model of Cox proportional hazard ratio was calculated by likelihood ratio test. (*p* < 0.0001).

^b^Other tumor sites included floor of mouth, buccal mucosa, mandible, maxilla, and lip.

^c^*p* < 0.05.

Among these genes, *GPAM* also showed significant prognostic relevance in univariate Cox analysis of TCGA mRNA dataset (*p* = 0.03; HR = 1.5). In the additional GEO RNA validation set, *CNPPD1* and *FLI1* exhibited significant expression in disease-specific and overall Cox analyses, respectively (*p* = 0.02 and 0.03; Table 2). *ZNF554, RAB2A,* and *SORCS2* showed borderline prognostic significance in this validation set (Table 2). These findings indicate the potential prognostic importance of these genes in OSCC, particularly in the context of PNI.

**Table 2:** Multivariate Cox regression models of prognostically significant RNA genes in HPV-negative oral squamous cell carcinoma patients (GSE41613 patients, N = 96).

Variable	TGFBR1^a^	RPS6KA4^a^	TYRO3^a^	GPR137	TEX10^a^	TPSD1^a^
	HR (*P*)	HR (*P*)	HR (*P*)	HR (*P*)	HR (*P*)	HR (*P*)
Gene expression	2.82 (0.02^b^)	3.45 (0.002^b^)	0.32 (0.006^b^)	0.42 (0.029^b^)	2.93 (0.004^b^)	2.32 (0.03^b^)
Age	0.57 (0.15)	0.60 (0.19)	0.88 (0.76)	1.28 (0.48)	0.86 (0.68)	0.61 (0.21)
Sex	1.18 (0.69)	1.20 (0.66)	1.51 (0.32)	1.18 (0.58)	1.05 (0.89)	1.32 (0.50)
AJCC stage						
Stage 1, 2 vs 3, 4	3.92 (0.02^b^)	5.64 (0.003^b^)	6.46 (0.002^b^)	5.29 (0.0001^b^)	4.55 (0.005^b^)	4.39 (0.02^b^)
Treatment						
Uni- vs multi-modality	1.01 (0.98)	1.00 (0.99)	0.87 (0.79)	0.65 (0.26)	0.74 (0.52)	0.88 (0.82)

Variable	CNPPD1^a^	FLI1^a^	RAB2A^a^	SORCS2^a^	ZNF554^a^	
	HR (*P*)	HR (*P*)	HR (*P*)	HR (*P*)	HR (*P*)	
Gene expression	0.40 (0.03^b^)	2.00 (0.02^b^)	0.51 (0.08)	1.75 (0.09)	0.42 (0.05)	
Age	0.59 (0.18)	1.20 (0.59)	0.83 (0.62)	0.80 (0.54)	0.64 (0.26)	
Sex	1.01 (0.98)	0.99 (0.98)	1.10 (0.80)	1.06 (0.86)	1.13 (0.77)	
AJCC stage						
Stage 1, 2 vs 3, 4	4.71 (0.009^b^)	5.24 (0.0001^b^)	5.81 (0.001^b^)	7.34 (0.0001^b^)	7.70 (0.001^b^)	
Treatment						
Uni- vs multi- modality	0.92 (0.87)	0.66 (0.27)	0.57 (0.23)	0.52 (0.14)	0.63 (0.34)	

*AJCC* American Joint Committee on Cancer.

^a^The statistical significance of fitted model of Cox proportional hazard ratio was calculated by likelihood ratio test. (*p* < 0.001).

^b^*p* < 0.05.

### Prognostic mRNA expressions associated with DNA mutations in perineural invasion

**Figure 2:**
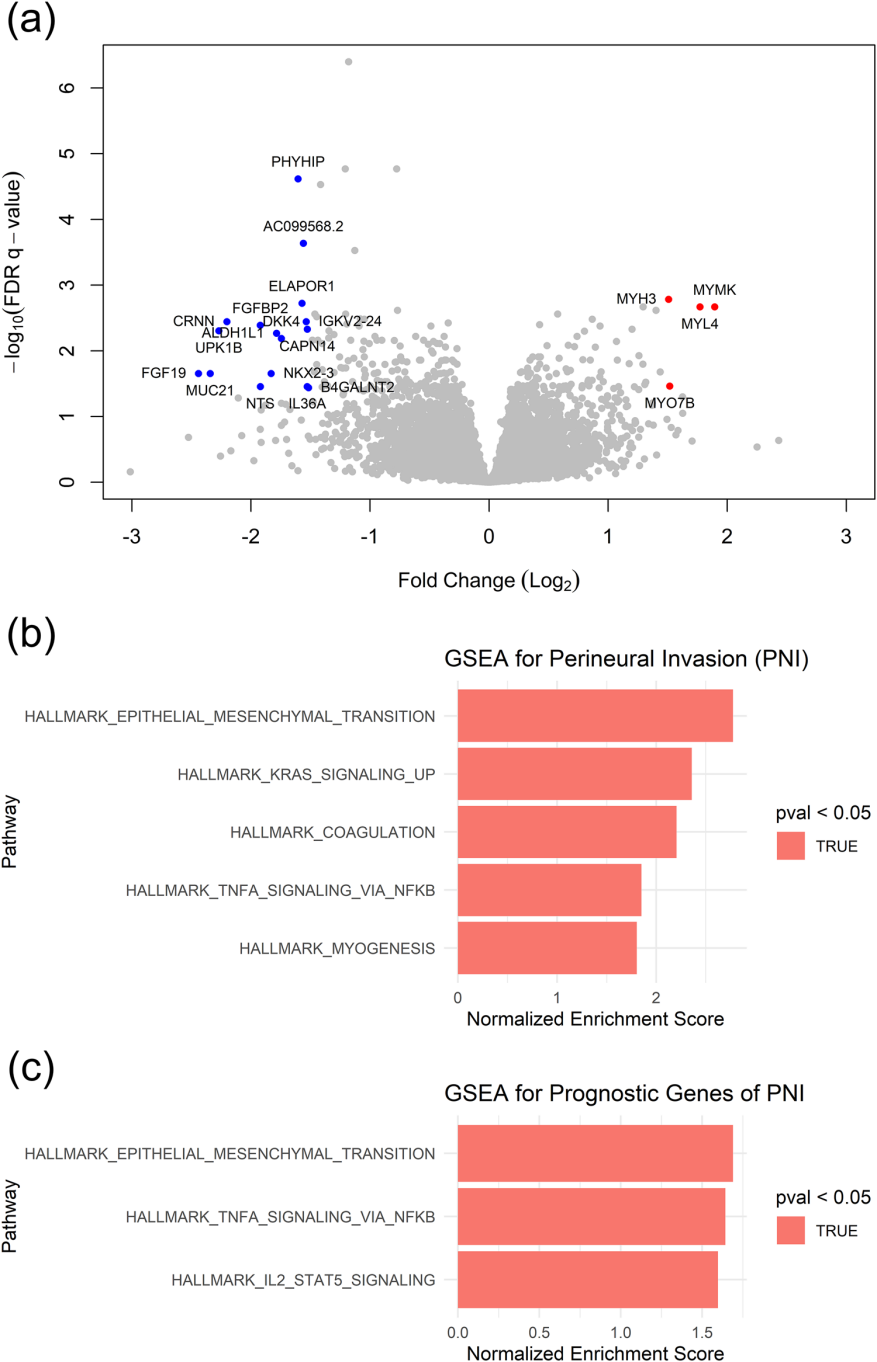
Differentially expressed mRNA genes (DEGs) and hallmark pathways with perineural invasion (PNI) in oral squamous cell carcinoma (OSCC). (**a**) DEGs associated with the presence or absence of PNI are illustrated in the volcano plot. Four genes (*MYH3, MYO7B, MYL4, MYMK*) exhibited significant upregulation, while 16 genes (*FGF19, MUC21, UPK1B, CRNN, FGFBP2, NTS, NKX2-3, ALDH1L1, DKK4, PHYHIP, ELAPOR1, AC099568.2, IGKV2-24, IL36A, CAPN14, B4GALNT2*) showed significant downregulation (|log2FC|> 1.5; FDR *q* < 0.05). (**b**) Hallmark pathways from gene set enrichment analyses exhibited significant differences in relation to PNI in OSCC. Upregulated hallmark pathways included epithelial-mesenchymal transition, KRAS signaling up, coagulation, TNFα signaling via NF-κB, and myogenesis. (**c**) Notably, prognostically significant genes in PNI demonstrated upregulation, encompassing hallmarks such as hallmarks epithelial-mesenchymal transition, TNFα signaling via NF-κB, and IL2 STAT5 signaling.

To investigate the relationship between gene mutation and mRNA expression in PNI of OSCC, mRNA expression levels between tumors with and without invasion were compared. A total of 20 DEGs from the two datasets was identified (|log2FC|> 1.5; FDR *q* < 0.05). Among these, four genes (*MYH3, MYO7B, MYL4, MYMK*) were significantly upregulated, while 16 genes (*FGF19, MUC21, UPK1B, CRNN, FGFBP2, NTS, NKX2-3, ALDH1L1, DKK4, PHYHIP, ELAPOR1, AC099568.2, IGKV2-24, IL36A, CAPN14, B4GALNT2*) were significantly downregulated after gene integration (|log2FC|> 1.5; FDR *q* < 0.05; Fig. 2a). The Normalized Enrichment Score (NES) from Gene Set Enrichment Analysis (GSEA) was employed to identify hallmark pathways significantly associated with PNI (DEGs FDR *q* < 0.05; Fig. 2b). Upregulated hallmark pathways include epithelial-mesenchymal transition, KRAS signaling up, coagulation, TNFα signaling via NF-κB, and myogenesis.

**Figure 3:**
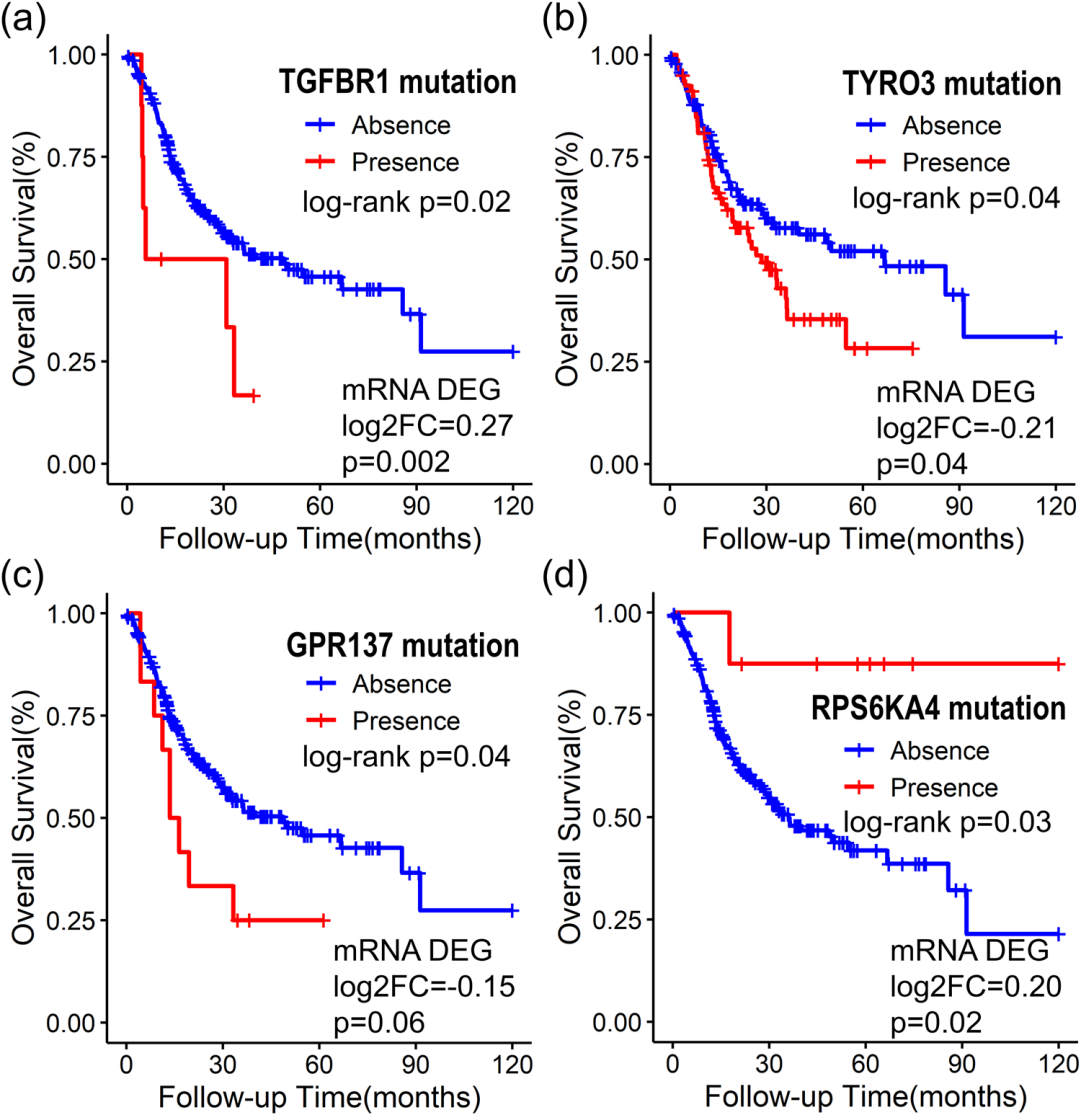
Kaplan–Meier survival curves illustrating the 10-year overall survival according to (**a**) *TGFBR1*, (**b**) *TYRO3*, (**c**) *GPR137*, and (**d**) *RPS6A4* mutations in oral squamous cell carcinoma with perineural invasion (PNI) (N = 224). The log2FC values and associated significant *p*-values of differentially expressed genes (DEGs) for each mRNA, based on the presence of PNI, were validated. *TGFBR1, TYRO3, RPS6KA4,* and *GPR137* not only exhibited significant mRNA expression in cases with PNI but also demonstrated significant prognostic implications in the validation set (Table 2).

Among the 22 DNA mutated genes with prognostic significance (Fig. 1 and Table S3), the mRNAs of *TGFBR1* and *RPS6KA4* were significantly overexpressed in PNI patients (log2FC = 0.27 and 0.20; *p* = 0.002 and 0.026, respectively; Fig. 3). Conversely, the mRNAs of *TYRO3* and *GPR137* were significantly underexpressed (log2FC = -0.21 and -0.14; *p* = 0.045 and 0.067, respectively; Fig. 3). Similar results were observed in additional GEO RNA validation sets. *TGFBR1* and *RPS6KA4* maintained prognostically significant expressions with HRs greater than 1 in multivariate Cox models for disease-specific survival (*p* = 0.02 and 0.002; Table 2). Conversely, *TYRO3* and *GPR137* demonstrated prognostically significant expressions with HRs less than 1 in disease-specific and overall Cox analyses, respectively (*p* = 0.006 and 0.029; Table 2). These findings suggest a potential link between the DNA mutations of these genes and their corresponding mRNA expressions in the context of PNI, highlighting their importance as prognostic indicators in OSCC.

In a previous comparative analysis, a total of 2801 differentially expressed genes were identified (*p* < 0.05). Out of these, univariate Cox analyses revealed that 245 genes were significantly associated with OSCC prognosis (*p* < 0.05). GSEA was also performed to identify hallmark pathways significantly associated with these 245 prognostic genes related to PNI (*p* < 0.05; Fig. 2c). The analysis revealed upregulated hallmark pathways, including epithelial-mesenchymal transition, TNFα signaling via NF-κB, and IL2 STAT5 signaling. Notably, *TGFB1*, which is related to *TGFBR1*, was a member of the GSEA hallmark epithelial-mesenchymal transition. GSEA was also performed to identify hallmark pathways significantly associated with these 245 prognostic genes related to PNI (*p* < 0.05; Fig. 2c). The analysis revealed upregulated hallmark pathways, including epithelial-mesenchymal transition, TNFα signaling via NF-κB, and IL2 STAT5 signaling. Notably, *TGFB1*, which is related to *TGFBR1*, was a member of the GSEA hallmark epithelial-mesenchymal transition.

Subsequently, the 245 prognostic significant genes underwent further adjusted for all major clinical variables using multivariate Cox regression. As a result, 141 genes displayed significant outcomes in multivariate Cox analyses. Among these 141 prognostic mRNA genes, *TPSD1, ZNF43, TEX10*, and *PSD3* (Table 3) also showed significant results in DNA frequency comparison analyses (*p* = 0.005, 0.011, 0.038, and 0.014, respectively). Notably, *TEX10* exhibited consistent results in additional GEO RNA validation sets (Table 2). However, *PSD3*, despite demonstrating prognostically significant expression, showed different HRs, indicating the need for additional research (Table 2).

**Table 3:** Multivariate Cox regression models of prognostically significant genes in mRNA expression associated with DNA mutations in perineural invasion of oral squamous cell carcinoma (The Cancer Genome Atlas patients, N = 218).

Variable	PSD3^a^		ZNF43^a^		TEX10^a^		TPSD1^a^	
	HR	*P*	HR	*P*	HR	*P*	HR	*P*
Gene	0.61	0.038^c^	0.61	0.04^c^	1.58	0.03^c^	0.56	0.049^c^
Age	1.02	0.02^c^	1.02	0.02^c^	1.02	0.02^c^	1.03	0.01^c^
Sex	1.05	0.85	0.98	0.93	1.01	0.96	1.07	0.78
Race								
White vs nonwhite	1.66	0.12	1.82	0.07	1.55	0.18	1.51	0.21
White vs unknown	1.36	0.57	1.25	0.68	1.24	0.68	1.20	0.73
Tumor site								
Tongue vs other sites^b^	1.18	0.52	1.09	0.75	1.00	1.00	1.09	0.74
Tongue vs unknown	1.44	0.21	1.29	0.36	1.13	0.66	1.25	0.42
AJCC stage								
Stage 1, 2 vs 3, 4	2.80	0.001^c^	2.27	0.01^c^	2.37	0.01^c^	2.38	0.01^c^
Lymphovascular invasion								
Absence vs presence	1.84	0.01^c^	1.93	0.01^c^	1.81	0.01^c^	1.81	0.01^c^
Absence vs unknown	0.92	0.81	0.96	0.91	0.90	0.77	0.88	0.73
Treatment								
Surgery vs surgery + RT	0.55	0.04^c^	0.49	0.02^c^	0.53	0.03^c^	0.49	0.02^c^
Surgery vs surgery + CCRT	0.74	0.31	0.69	0.21	0.72	0.26	0.66	0.16
Surgery vs unknown	2.77	0.01	1.92	0.07	1.88	0.08	2.03	0.05

*AJCC* American Joint Committee on Cancer, *RT* radiotherapy, *CCRT* concurrent chemotherapy and radiotherapy, *HR* hazard ratio.

^a^The statistical significance of fitted model of Cox proportional hazard ratio was calculated by likelihood ratio test. (*p* < 0.0001).

^b^Other tumor sites included floor of mouth, buccal mucosa, mandible, maxilla, and lip.

^c^*p* < 0.05.

**Figure 4:**
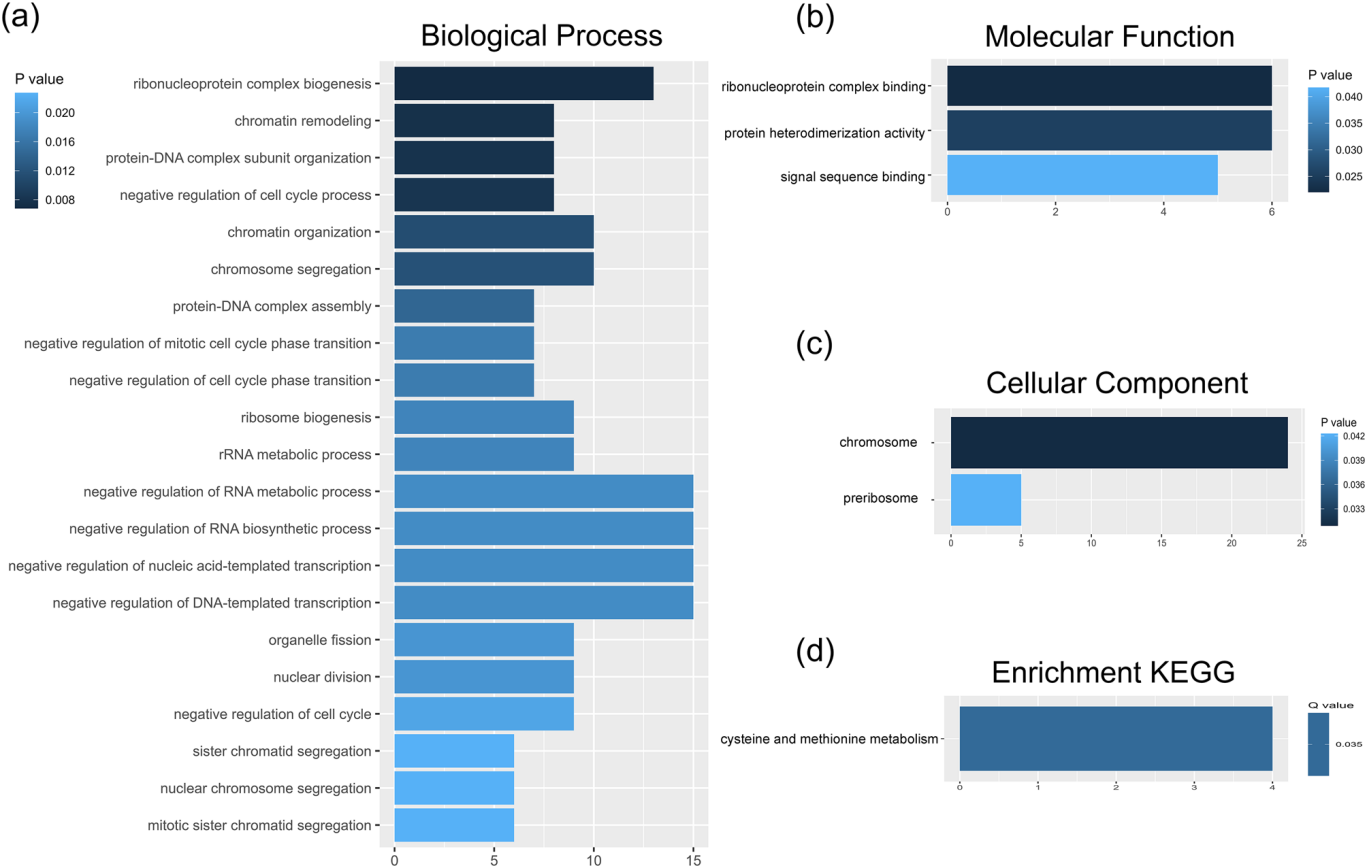
Results of Gene Ontology (GO) enrichment analysis for genes with significant prognostic implications associated with perineural invasion in oral squamous cell carcinoma. The analysis encompasses (**a**) biological processes, (**b**) molecular functions, and (**c**) cellular components. Additionally, (**d**) KEGG pathway enrichment analysis is provided. These analyses are presented based on their statistical significance.

The 141 prognostic significant genes from the multivariate Cox regression were then subjected to Gene Ontology (GO) and KEGG pathway enrichment analyses (Fig. 4). In the GO enrichment analyses for significant prognostic PNI genes, biological processes, molecular functions, and cellular components were examined (Fig. 4a–c). Additionally, the significant KEGG pathway hsa00270 cysteine and methionine metabolism (FDR *q* = 0.035, Fig. 4d) was investigated, and the locations of differentially expressed mRNA genes (*p* < 0.01; Fig. S1a) were analyzed.

### Amino acid alterations and biological pathways in prognostic DNA and mRNA profiles

**Figure 5:**
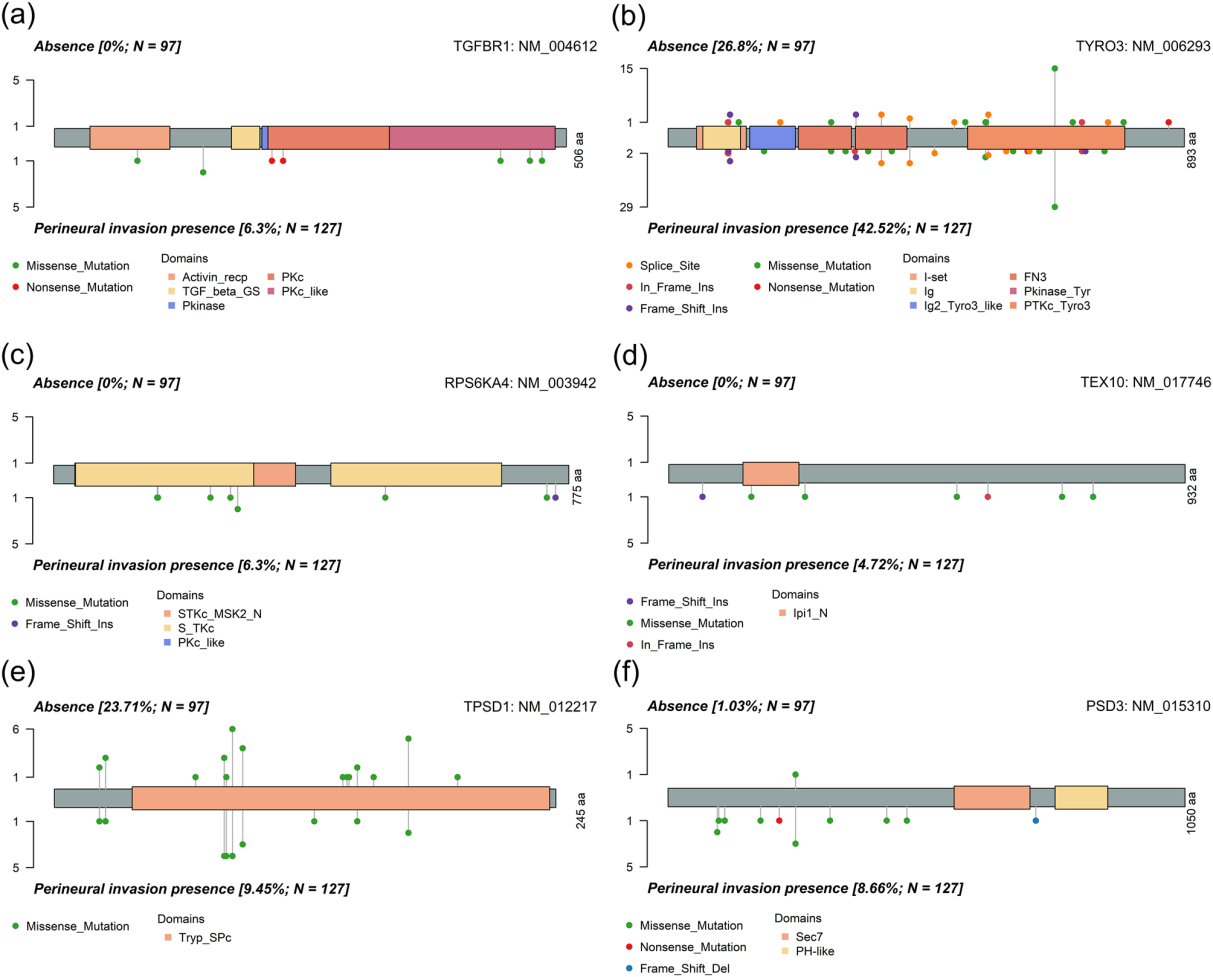
Amino acid alterations of genes with significant prognostic implications across DNA mutations and mRNA expression in oral squamous cell carcinoma patients with perineural invasion. (**a**) *TGFBR1*, (**b**) *TYRO3*, (**c**) *RPS6KA4* depict genes that exhibit substantial prognostic implications and demonstrate significant differences in DNA mutations related to PNI. Amino acid alterations in genes with significantly differentially expressed mRNA are illustrated for these genes. Similarly, (**d**) *TEX10*, (**e**) *TPSD1*, and (**f**) *PSD3* represent genes displaying notable prognostic implications among those with significantly differentially expressed mRNA in relation to PNI. For this subset, the figure illustrates the amino acid changes in genes that exhibited significant differences in DNA mutations.

Within the DNA mutations exhibiting significant differences related to PNI, genes bearing substantial prognostic implications were identified through univariate Cox analyses (Table S3). Among these genes, the amino acid changes of significantly differentially expressed mRNA genes were examined (Fig. 5a–c). Additionally, genes with notable prognostic implications in both univariate and multivariate Cox analyses among the significantly differentially expressed mRNA genes associated with PNI were identified. For this subset, we further explored the amino acid changes in genes that exhibited significant differences in DNA mutations linked to PNI (Fig. 5d–f).

To identify biological pathways closely associated with OSCC prognosis, KEGG analysis encompassing all prognostically significant DNA and mRNA genes (Table S5) was conducted. Two genes were associated with each of the following pathways: hsa04144 endocytosis (*TGFBR1, PSD3*), hsa04010 MAPK signaling pathway (*TGFBR1, RPS6KA4*), and hsa01100 metabolic pathways (*GPAM, GGCX*). We assessed the involvement of mRNA differential expression genes (*p* < 0.01; Fig. S1b, c) in the hsa04144 endocytosis and hsa04010 MAPK signaling pathways. Additionally, the online tool DAVID was used to perform GO enrichment analysis of significant prognostic genes. The genes were clustered according to the GO biological process terms as follows: “GO:0007399 ~ nervous system development” (*ADGRG1, PCDHGC4, TYRO3, TGFBR1*), “GO:0006355 ~ regulation of transcription, DNA-templated” (*RPS6KA4, ZNF43, ZNF699, ZNF554, TGFBR1*), “GO:0050431 ~ transforming growth factor beta binding” (*TGFBR1, VASN*) and “GO:0016021 ~ integral component of membrane” (*POTEM, ADGRG1, GGCX, GPAM, PCDHGC4, CLEC6A, TYRO3, GPR137, SORCS2, CNPPD1, TGFBR1, VASN*; Table S6).

## Discussion

In the present study, we compared the genomes and transcriptomes of OSCC tumors in relation to PNI and OS and validated this finding using GEO datasets. Among the DNA mutations that were found to be significant in PNI and showed significance in univariate Cox regression, *TGFBR1, TYRO3, RPS6KA4,* and *GPR137* displayed significant differential mRNA expression. Additionally, among the mRNA genes that exhibited significant differential expression in PNI and were significant in multivariate Cox regression, *TPSD1, ZNF43, TEX10,* and *PSD3* demonstrated significant differential DNA mutations. Among them, six genes (*TGFBR1, RPS6KA4, TYRO3, GPR137, TEX10* and *TPSD1*) were validated using mRNA prognostic data.

In DNA mutation analysis, twenty-two genes (*ANKRD18B, ZNF699, UBE2E3, ZNF554, VASN, IGKV1-17, CLEC6A, PCDHGC4, GPAM, SORCS2, FLI1, ADGRG1, PSD3, TGFBR1, GGCX, IGKV1-16, RAB2A, TYRO3, GPR137, RPS6KA4, CNPPD1,* and *POTEM*) were related to both PNI and OS. Interestingly, four of the 22 genes (*ADGRG1, PCDHGC4, TYRO3,* and *TGFBR1*) clustered according to the functional terms of both “integral component of membrane” and “nervous system development” as shown in Table S5. Similarly, Saidak et al. examined the list of mRNA genes associated with PNI and identified genes with a well-established function in neural differentiation/neuritogenesis, such as *NGF* in HNSCC^6^. Considering genomic analysis about PNI, Koo et al. found significant association with EGFR amplification in OSCC of non-smoking and non-drinking patients^13^. Our RNAseq results also showed significant differential expression of *EFGR* (*p* = 0.03, |log2FC|= 0.38) and genes related to EGF pathway (*PRKCA, JAK1, PLCG1, p* = 0.01, 0.02, 0.02 and |log2FC|= 0.32, 0.20, 0.16, respectively) in PNI patients, although prognosis was not significant. Higher frequency of *KRAS* mutations were found in intrahepatic cholangiocarcinoma patients and colon cancer with PNI^14,15^, coincide with RNAseq results in Fig. 2b. Among genes up-regulated by hallmark KRAS signaling in GSEA, *CSF2, PCSK1N, MMP10, MMP11, SCG5* and *INHBA* were the leading edge genes. Also, *IL33* shows significant relation to prognosis in multivariate Cox analysis in KRAS pathway (*p* = 0.004, HR = 2.02).

DEG analysis revealed that prognostic mRNA of PNI were related to hallmarks epithelial mesenchymal transition, TNFα signaling via NF-κB, and IL2 /STAT5 signaling in GSEA analysis and ribonucleoprotein complex biogenesis, chromatin remodeling, protein-DNA complex subunit organization, and cysteine and methionine metabolism etc. in GO and KEGG analysis. Especially, the hallmarks epithelial mesenchymal transition pathway overlapped with the DNA mutation mentioned above, *TGFBR1*. EMT is a process by which polar epithelial cells are transformed into mesenchymal cells with a significantly enhanced ability to migrate and invade the surrounding tissues and already well known for its relationship with PNI in HNSCC^7,16^. There were strong expression of BDNF, TrkB, and p75NGFR in perineural tumor cells of human cutaneous squamous cell carcinoma^17^, and over-expression of TrkB results in altered expression of molecular mediators of EMT, including downregulation of E-cadherin and upregulation of Twist in HNSCC^18^. Considering TNFα signaling via NF-κB, TNFα promotes oral cancer proliferation, progression, and nociception at least partially by activating Schwann cells^19^. IL2 /STAT5 signaling is a key regulator of CD4^+^T cell gene program and known to enrich in low risk group of HNSCC^20,21^. GO and KEGG analysis results were broadly associated with maintenance, translation and mitosis of DNA and RNA. However, key genes and pathways in our study was different from the previous studies. Zhang et al. evaluated the transcriptomes of PNI in HNSCC using weighted gene coexpression network analysis and found 12 genes (*TIMP2, MIR198, LAMA4, FAM198B, MIR4649, COL5A1, COL1A2, OLFML2B, MMP2, FBN1, ADAM12,* and *PDGFRB*) were highly expressed in fibroblasts^7^. Otherwise, Saidak et al. identified a specific gene expression profile highly enriched in genes related to muscle differentiation/ function in HNSCC^6^. These differences seemed to have originated from our study’s assignment of equal importance to both survival and PNI. PNI is a well-known risk factors in the prognosis of OSCC and we want to identify the key genes that could influence both survival and PNI.

We identified eight overlapping genes, *TGFBR1, RPS6KA4, TYRO3, GPR137, ZNF43, TEX10* and *TPSD1* and *PSD3*, that exhibited both DNA mutations and differential mRNA expressions regarding PNI. *TGFBR1* and *TEX10* was a risk gene in DNA survival analysis and overexpressed in PNI cases. *Transforming growth factor-β receptor type 1* (*TGFBR1*) is a receptor for TGF-β ligands that modulates cancer stem cell properties and epithelial-mesenchymal transition. *TGFBR1* is a crucial inducer of lung cancer progression, and inhibition of *TGFBR1* effectively reduces bone metastasis in prostate cancer^22^. Regarding neurogenesis, TGF-β is a major extracellular signaling molecule in neuronal cells during CNS angiogenesis, mediating the recruitment of endothelial TGFBR1 to regulate endothelial proliferation and cerebrovascular integrity ^23^. *Testis expressed 10* (*Tex10*) has a role in transcriptional regulation, ribosome biogenesis, the establishment and maintenance of pluripotency and is known to be upregulated, promoting cancer stem cell properties and therapy resistance in hepatocellular carcinoma, esophageal SCC and urinary bladder carcinoma ^24,25^. *Ribosomal Protein S6 Kinase A4* (*RPS6KA4)*, identified as a non-risk gene in DNA survival analysis and found to be overexpressed in PNI cases, belongs to the ribosomal S6 kinase family. It acts as a downstream effector of MAPK pathway, phosphorylating substrates that are involved in cell growth, proliferation, motility and survival. In a study involving hepatocellular carcinoma patients, those with higher expression levels of *RPS6KA4* exhibited a poorer survival ^26^. *TYRO3* and *GPR137* were a risk gene and under-expressed in PNI patients. *TYRO3* is a member of the TAM (TYRO3, AXL, and MERTK) family of transmembrane receptor tyrosine kinases, which have been implicated in tumorigenesis, metastasis and therapeutic resistance^27^. Tumors exhibiting high *TYRO3* expression often display resistance in patients receiving anti-PD-1/PD-L1 therapy. Inhibition of *TYRO3* promotes tumor ferroptosis and sensitizes resistant tumors to anti-PD-1 therapy^28^. Concerning neurogenesis, the *tyro3* gene is known to be expressed during central nervous system neurogenesis and displays distinct and highly regionalized patterns of expression in the adult brain^29^. However, TCGA mRNA expression study revealed underexpression in PNI patients and HR lower than 1 in GEO data. This discrepancy warrants further focused study regarding mechanisms. *G protein-coupled receptor 137 (GPR137)* has been recognized as a key player in carcinogenesis and cancer progression, and its down regulation has been shown to inhibit proliferation and promote apoptosis in leukemia cells. Patients with high *GPR137* expression exhibited shorter overall survival time than those with low expression in bladder cancer ^30,31^. However, other studies reported a different role for *GPR137,* where it promotes cell cycle exit and neuronal differentiation in neuro2A cells ^32^. Our study’s finding of under-expression of *GPR137* in PNI patients may be associated with this characteristic. These 5 genes were differentially expressed in GEO data with similar expression pattern. These results indicate a possible association between the DNA mutations of these genes and their corresponding mRNA expressions in relation to PNI, underscoring their significance as markers for prognosis in OSCC. *ZNF43* and *TPSD1* were identified as nonhazardous genes and found to be underexpressed in PNI patients. *Zinc finger protein 43* (*ZNF43*) is known to be involved in transcriptional regulation and is classified as a tumor-suppressor gene. Methylation at specific CpGsites of *ZNF43* was known to indicates aggressive behavior^33^. However, our validation did not reveal the significance and further clinical corroboration would be necessary. *Tryptase Delta 1* (*TPSD1*) belongs to a family of trypsin-like serine proteases and is thought to play a role in the regulation of mRNA stability. A specific mutation in *TPSD1* was observed in colon cancer patients who did not respond well to chemotherapy^34^. Our GEO RNA validation result were not consistent with TCGA mRNA result with different HR, indicating the need for additional research. *Pleckstrin And Sec7 Domain Containing 3* (*PSD3)* was a risk gene and its expression also affect the patient’s survival. *PSD3* acted as a potential biomarker predicting relapse of acute myeloid leukemia in cytogenetically normal adult patients and involved in breast cancer metastasis, astrocytoma and papillary thyroid cancer progression ^35^.

This study has the limitation. While we conducted an analysis by comparing somatic mutation data with mRNA expression data, it is important to acknowledge that the identified genes have only been validated using prognostic data. Nevertheless, our bioinformatics-driven approach in identifying key genes and pathways will provides insights for prospective research endeavors.

## Conclusion

In summary, through a comparative analysis of prognosis and PNI between DNA mutation and mRNA expression data of TCGA, we identified eight overlapping genes, *TGFBR1, RPS6KA4, TYRO3, GPR137, ZNF43, TEX10, TPSD1,* and *PSD3*, associated with PNI in OSCC. Prognostic DNA mutations clustered as the following terms: nervous system development (*ADGRG1, PCDHGC4, TYRO3, TGFBR1*), regulation of DNA-templated transcription (*RPS6KA4, ZNF43, ZNF699, ZNF554, TGFBR1*), and transforming growth factor beta binding (*TGFBR1, VASN*). Prognostic mRNA expression analysis showed the upregulation of the epithelial mesenchymal transition pathway. Six genes (*TGFBR1, RPS6KA4, TYRO3, GPR137, TEX10* and *TPSD1*) were validated using prognostic data. Considering that PNI serves as an independent risk factor for poor prognosis, our findings provide a valuable perspective for the development of novel treatment strategies tailored to OSCC patients with PNI.

### Supplementary Information

Supplementary Figure S1.

Supplementary Tables.

## Data Availability

The data that support the findings of this study are available from the Cancer Genome Atlas (http://gdc.cancer.gov/, accession number: phs000178.v11.p8) and GSE41613 (https://www.ncbi.nlm.nih.gov/geo/query/acc.cgi?acc=GSE41613).
