# Supplementary figures and images for: Prognostic DNA mutation and mRNA expression analysis of perineural invasion in oral squamous cell carcinoma
Source: Sci Rep. 2024 Jan 29;14:2427. doi: 10.1038/s41598-024-52745-6 (PMC10825128; doi:10.1038/s41598-024-52745-6)

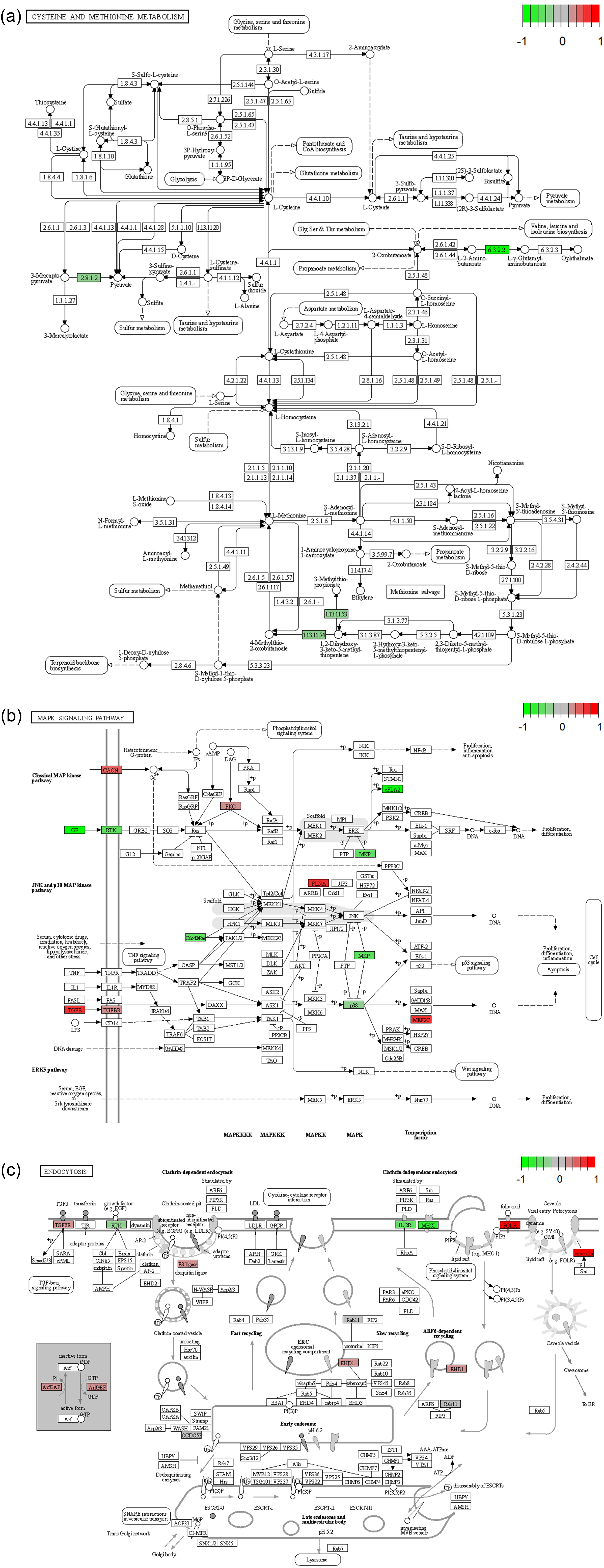

Supplement: Supplementary file 1 — Supplementary Figure S1. [file 41598_2024_52745_MOESM1_ESM.tiff]
