# Supplementary material for: Prognostic DNA mutation and mRNA expression analysis of perineural invasion in oral squamous cell carcinoma
Source: Sci Rep. 2024 Jan 29;14:2427. doi: 10.1038/s41598-024-52745-6 (PMC10825128; doi:10.1038/s41598-024-52745-6)
Supplement: Supplementary file 2 — Supplementary Tables. [file 41598_2024_52745_MOESM2_ESM.docx]

**Supplementary Figure**

**Figure S1** In the Kyoto Encyclopedia of Genes and Genomes (KEGG) analysis involving prognostically significant genes related to perineural invasion: (a) The hsa00270 cysteine and methionine metabolism pathway (FDR *q* = 0.035) was identified. (b) The hsa04010 MAPK signaling pathway, featuring genes *TGFBR1* and *RPS6KA4*, was identified. (c) The hsa04144 endocytosis pathway, involving genes *TGFBR1* and *PSD3*, was identified. Within these three mechanisms, we examined the participation of mRNA differential expression genes (*p* < 0.01).

**Supplementary Tables**

**Table S1**. Clinicopathological data of The Cancer Genome Atlas patients.

| **Perineural invasion** | |  | **Absence** | |  | **Presence** | |  | **Total** | |
| --- | --- | --- | --- | --- | --- | --- | --- | --- | --- | --- |
| Variable | |  | No. | % |  | No. | % |  | No. | % |
| **No. of patients** | |  | 97 | 43.3 |  | 127 | 56.7 |  | 224 | |
| **Age(yrs.)** ^a^ | |  | 61(24-87) | |  | 61(19-90) | |  | 61(19-90) | |
| **Sex** | |  |  |  |  |  |  |  |  |  |
|  | Male |  | 66 | 68.0 |  | 87 | 68.5 |  | 153 | 68.3 |
|  | Female |  | 31 | 32.0 |  | 40 | 31.5 |  | 71 | 31.7 |
| **Race** | |  |  |  |  |  |  |  |  |  |
|  | White |  | 82 | 84.5 |  | 108 | 85.0 |  | 190 | 84.8 |
|  | Nonwhite |  | 12 | 12.4 |  | 14 | 11.0 |  | 26 | 11.6 |
|  | Unknown |  | 3 | 3.1 |  | 5 | 4.0 |  | 8 | 3.6 |
| **Primary tumor site** | |  |  |  |  |  |  |  |  |  |
|  | Tongue |  | 33 | 34.0 |  | 54 | 42.5 |  | 87 | 38.8 |
|  | Floor of mouth |  | 26 | 26.8 |  | 25 | 19.7 |  | 51 | 22.8 |
|  | Buccal mucosa |  | 8 | 8.3 |  | 13 | 10.2 |  | 21 | 9.4 |
|  | Mandible and maxilla |  | 9 | 9.3 |  | 9 | 7.1 |  | 18 | 8.0 |
|  | Lip |  | 0 | 0.0 |  | 1 | 0.8 |  | 1 | 0.5 |
|  | Mouth, unknown |  | 21 | 21.6 |  | 25 | 19.7 |  | 46 | 20.5 |
| **AJCC stage** | |  |  |  |  |  |  |  |  |  |
|  | Stage 1 and 2 |  | 30 | 30.9 |  | 18 | 14.2 |  | 48 | 21.4 |
|  | Stage 3 and 4 |  | 67 | 69.1 |  | 109 | 85.8 |  | 176 | 78.6 |
| **Lymphovascular invasion** | |  |  |  |  |  |  |  |  |  |
|  | Absence |  | 75 | 77.3 |  | 64 | 50.4 |  | 139 | 62.0 |
|  | Presence |  | 19 | 19.6 |  | 45 | 35.4 |  | 64 | 28.6 |
|  | Unknown |  | 3 | 3.1 |  | 18 | 14.2 |  | 21 | 9.4 |
| **Treatment modality** | |  |  |  |  |  |  |  |  |  |
|  | Surgery |  | 37 | 38.1 |  | 34 | 26.8 |  | 71 | 31.7 |
|  | Surgery+RT |  | 29 | 29.9 |  | 36 | 28.3 |  | 65 | 29.0 |
|  | Surgery+CCRT |  | 22 | 22.7 |  | 47 | 37.0 |  | 69 | 30.8 |
|  | Unknown |  | 9 | 9.3 |  | 10 | 7.9 |  | 19 | 8.5 |

Abbreviations: AJCC, American Joint Committee on Cancer; RT, radiotherapy; CCRT, concurrent chemotherapy and radiotherapy.

a median (range) value

**Table S2**. Clinicopathological data of GSE41613 patients.

| **No. of patients** | |  | 96 | % |
| --- | --- | --- | --- | --- |
| **Age(yrs.)** | |  |  |  |
|  | <50 |  | 22 | 22.9 |
|  | 50≤ |  | 74 | 77.1 |
| **Sex** | |  |  |  |
|  | Male |  | 65 | 67.7 |
|  | Female |  | 31 | 32.3 |
| **HPV state** | |  |  |  |
|  | Negative |  | 96 | 100 |
|  | Positive |  | 0 | 0 |
| **AJCC stage** | |  |  |  |
|  | Stage 1 and 2 |  | 41 | 42.7 |
|  | Stage 3 and 4 |  | 55 | 57.3 |
| **Treatment modality** | |  |  |  |
|  | Uni-modality |  | 43 | 44.8 |
|  | Multi-modality |  | 53 | 55.2 |

Abbreviations: AJCC, American Joint Committee on Cancer; HPV, human papilloma virus

**Table S3**. Univariate and multivariate Cox regression models of major clinicopathological variables in oral squamous cell carcinoma cases (The Cancer Genome Atlas patients, N = 224).

|  | | | **Univariate analysis** | | | | **Multivariate analysis**^a^ | | | |
| --- | --- | --- | --- | --- | --- | --- | --- | --- | --- | --- |
| variable | | | HR | 95%CI | | *P* | HR | 95%CI | | *P* |
| **Age** | | | 1.02 | 1.00-1.03 | | 0.07 | 1.02 | 1.00-1.04 | | 0.05 |
| **Sex** | | | 0.93 | 0.62-1.40 | | 0.72 | 0.94 | 0.60-1.47 | | 0.79 |
| **Race** | | |  |  |  |  |  |  |  |  |
|  | white vs nonwhite | | 1.43 | 0.78-2.62 | | 0.25 | 1.64 | 0.86-3.11 | | 0.13 |
|  | white vs unknown | | 1.00 | 0.36-2.72 | | 0.99 | 1.11 | 0.39-3.17 | | 0.85 |
| **Tumor site** | | |  |  |  |  |  |  |  |  |
|  | tongue vs other sites^b^ | | 1.20 | 0.76-1.88 | | 0.44 | 1.16 | 0.71-1.90 | | 0.56 |
|  | tongue vs unknown | | 1.41 | 0.84-2.35 | | 0.19 | 1.25 | 0.73-2.13 | | 0.41 |
| **AJCC stage** | | |  |  |  |  |  |  |  |  |
|  | | stage 1,2 vs stage 3,4 | 2.27 | 1.28-4.00 | | 0.005^c^ | 2.32 | 1.26-4.28 | | 0.007^c^ |
| **Perineural invasion** | | |  |  |  |  |  |  |  |  |
|  | | absence vs presence | 1.79 | 1.17-2.73 | | 0.007^c^ | 1.62 | 1.03-2.54 | | 0.03^c^ |
| **Lymphovascular invasion** | | |  |  |  |  |  |  |  |  |
|  | | absence vs presence | 1.71 | 1.13-2.60 | | 0.01^c^ | 1.64 | 1.04-2.57 | | 0.03^c^ |
|  | | absence vs unknown | 0.90 | 0.46-1.76 | | 0.76 | 0.78 | 0.39-1.58 | | 0.50 |
| **Treatment modality** | | |  |  |  |  |  |  |  |  |
|  | surgery vs surgery + RT | | 0.68 | 0.40-1.15 | | 0.15 | 0.53 | 0.30-0.93 | | 0.03^c^ |
|  | surgery vs surgery + CCRT | | 1.00 | 0.61-1.63 | | 0.99 | 0.68 | 0.39-1.21 | | 0.19 |
|  | surgery vs unknown | | 1.90 | 1.01-3.57 | | 0.05 | 1.96 | 0.99-3.92 | | 0.06 |

Abbreviations: AJCC, American Joint Committee on Cancer; RT, radiotherapy; CCRT, concurrent chemotherapy and radiotherapy; HR, hazard ratio; CI, confidence interval.

a The statistical significance of fitted model of Cox proportional hazard ratio was calculated by Likelihood ratio test. (*p* = 0.00009)

b Other tumor sites included floor of mouth, buccal mucosa, mandible, maxilla, and lip.

c *p* < 0.05

**Table S4**. Significant genes of perineural invasion and gene prognosis analyses in oral squamous cell carcinoma cases (*p* < 0.05; The Cancer Genome Atlas patients, N = 224).

| **Hugo Symbol** | **Perineural**  **Invasion** | | **Fisher’s**  **exact test**  *p*-value^a^ | **Univariate**  **Cox** **analysis**  *p*-value^a^ | | **Hazard Ratio** |
| --- | --- | --- | --- | --- | --- | --- |
|  | **Absent** (no.) | **Present** (no.) |  |  |  |  |
| ANKRD18B | 1 | 9 | 0.04 | 0.00004^b^ | 3.65 | |
| ZNF699 | 0 | 8 | 0.01 | 0.0002^b^ | 3.83 | |
| UBE2E3 | 0 | 6 | 0.04 | 0.0007^b^ | 3.81 | |
| ZNF554 | 0 | 7 | 0.02 | 0.002^b^ | 3.40 | |
| VASN | 0 | 6 | 0.04 | 0.003^b^ | 3.35 | |
| IGKV1-17 | 8 | 24 | 0.03 | 0.003^b^ | 2.06 | |
| CLEC6A | 0 | 6 | 0.04 | 0.01 | 3.04 | |
| PCDHGC4 | 0 | 12 | 0.001^b^ | 0.01 | 2.39 | |
| GPAM | 3 | 14 | 0.04 | 0.01 | 2.16 | |
| SORCS2 | 1 | 11 | 0.01 | 0.01 | 2.46 | |
| ADGRG1 | 0 | 6 | 0.04 | 0.02 | 2.78 | |
| TGFBR1 | 0 | 8 | 0.01 | 0.02 | 2.55 | |
| GGCX | 0 | 6 | 0.04 | 0.02 | 3.01 | |
| FLI1 | 0 | 12 | 0.001^b^ | 0.03 | 2.09 | |
| PSD3 | 1 | 11 | 0.01 | 0.03 | 1.95 | |
| RAB2A | 0 | 8 | 0.01 | 0.03 | 2.27 | |
| IGKV1-16 | 13 | 38 | 0.004^b^ | 0.03 | 1.58 | |
| TYRO3 | 26 | 54 | 0.02 | 0.04 | 1.52 | |
| GPR137 | 0 | 12 | 0.001^b^ | 0.04 | 2.00 | |
| RPS6KA4 | 0 | 8 | 0.01 | 0.02 | 0.13 | |
| CNPPD1 | 20 | 9 | 0.004^b^ | 0.01 | 0.37 | |
| POTEM | 7 | 2 | 0.04 | 0.04 | 0.17 | |

a *p* < 0.05

b *p* < 0.01

**Table S5**. Kyoto Encyclopedia of Genes and Genomes (KEGG) analysis of prognostic significant genes.

| **Genes** | **Pathways** |
| --- | --- |
| TGFBR1 | hsa04010 MAPK signaling pathway, hsa04659 Th17 cell differentiation, hsa05415 Diabetic cardiomyopathy, hsa04390 Hippo signaling pathway, hsa05161 Hepatitis B, hsa05142 Chagas disease, hsa05226 Gastric cancer, hsa05200 Pathways in cancer, hsa05166 Human T-cell leukemia virus 1 infection, hsa05210 Colorectal cancer, hsa05225 Hepatocellular carcinoma, hsa04380 Osteoclast differentiation, hsa04060 Cytokine-cytokine receptor interaction, hsa05220 Chronic myeloid leukemia, hsa04068 FoxO signaling pathway, hsa04218 Cellular senescence, hsa04933 AGE-RAGE signaling pathway in diabetic complications, hsa04371 Apelin signaling pathway, hsa04520 Adherens junction, hsa04350 TGF-beta signaling pathway, hsa04144 Endocytosis, hsa05212 Pancreatic cancer, hsa04926 Relaxin signaling pathway |
| TYRO3 | hsa03264 Virion - Flavivirus |
| RPS6KA4 | hsa04668 TNF signaling pathway, hsa04010 MAPK signaling pathway |
| TPSD1 | hsa05164 Influenza A |
| ZNF43 | hsa05168 Herpes simplex virus 1 infection |
| TEX10 | hsa03083 Polycomb repressive complex |
| PSD3 | hsa04144 Endocytosis |
| ZNF699 | hsa05168 Herpes simplex virus 1 infection |
| UBE2E3 | hsa04120 Ubiquitin mediated proteolysis |
| ZNF554 | hsa05168 Herpes simplex virus 1 infection |
| GPAM | hsa00561 Glycerolipid metabolism,  hsa00564 Glycerophospholipid metabolism, hsa01100 Metabolic pathways |
| FLI1 | hsa05202 Transcriptional misregulation in cancer |
| RAB2A | hsa04152 AMPK signaling pathway |
| CLEC6A | hsa04625 C-type lectin receptor signaling pathway |
| GGCX | hsa01240 Biosynthesis of cofactors, hsa01100 Metabolic pathways,  hsa00130 Ubiquinone and other terpenoid-quinone biosynthesis |

**Table S6**. Gene ontology enrichment analysis of prognostic significant genes.

| **Category** | **Term** | **No.** | ***P*** | **Genes** | **Fold  Enrichment** |
| --- | --- | --- | --- | --- | --- |
| GOTERM BP_DIRECT | GO:0007399~  nervous system development | 4 | 0.01^a^ | ADGRG1, PCDHGC4,  TYRO3, TGFBR1 | 8.44 |
| GOTERM BP_DIRECT | GO:0006355~  regulation of transcription,  DNA-templated | 5 | 0.02^a^ | RPS6KA4, ZNF43, ZNF699, ZNF554, TGFBR1 | 4.39 |
| GOTERM CC_DIRECT | GO:0016021~  integral component  of membrane | 12 | 0.02^a^ | POTEM, ADGRG1, GGCX,  GPAM, PCDHGC4, CLEC6A, TYRO3, GPR137, SORCS2, CNPPD1, TGFBR1, VASN | 1.91 |
| GOTERM MF_DIRECT | GO:0050431~  transforming growth factor beta binding | 2 | 0.03^a^ | TGFBR1, VASN | 68.89 |

Abbreviations: GO, gene ontology.

a *p* < 0.05
